# The use of tail-anchored protein chimeras to enhance liposomal cargo delivery

**DOI:** 10.1371/journal.pone.0212701

**Published:** 2019-02-22

**Authors:** Abbi Abdelrehim, Lior Shaltiel, Ling Zhang, Yechezkel Barenholz, Stephen High, Lynda K. Harris

**Affiliations:** 1 Faculty of Biology, Medicine and Health, University of Manchester, Manchester, United Kingdom; 2 Lipocure Ltd., Jerusalem, Israel; 3 Membrane and Liposome Research Lab, Hadassah Medical School of the Hebrew University, Jerusalem, Israel; 4 Division of Pharmacy and Optometry, Faculty of Biology, Medicine and Health, University of Manchester, Manchester, United Kingdom; 5 Maternal and Fetal Health Research Centre, Institute of Human Development, University of Manchester, Manchester, United Kingdom; 6 St. Mary's Hospital, Central Manchester University Hospitals NHS Foundation Trust, Manchester Academic Health Science Centre, Manchester, United Kingdom; Catholic University of Korea, REPUBLIC OF KOREA

## Abstract

**Background:**

Liposomes are employed as drug delivery vehicles offering a beneficial pharmacokinetic/distribution mechanism for in vivo therapeutics. Therapeutic liposomes can be designed to target specific cell types through the display of epitope-specific targeting peptides on their surface. The majority of peptides are currently attached by chemical modification of lipid constituents. Here we investigate an alternative and novel method of decorating liposomes with targeting ligand, using remotely and spontaneously inserting chimeric tail-anchored membrane (TA) proteins to drug loaded liposomes.

**Methods and results:**

An artificial TA protein chimera containing the transmembrane domain from the spontaneously inserting TA protein cytochrome b5 (Cytb5) provided a robust membrane tether for the incorporation of three different targeting moieties into preformed liposomes. The moieties investigated were the transactivator of transcription (TAT) peptide, the EGF-receptor binding sequence GE11 and the placental and tumour homing ligand CCGKRK. In all cases, TA protein insertion neither significantly altered the size of the liposomes nor reduced drug loading. The efficacy of this novel targeted delivery system was investigated using two human cell lines, HeLa M and BeWo. Short term incubation with one ligand-modified TA chimera, incorporating the TAT peptide, significantly enhanced liposomal delivery of the encapsulated carboxyfluorescein reporter.

**Conclusion:**

The Cytb5 TA was successfully employed as a membrane anchor for the incorporation of the desired peptide ligands into a liposomal drug delivery system, with minimal loss of cargo during insertion. This approach therefore provides a viable alternative to chemical conjugation and its potential to accommodate a wider range of targeting ligands may provide an opportunity for enhancing drug delivery.

## Introduction

Encapsulation of cytotoxic chemotherapeutics in liposomes improves their bioavailability and reduces their toxicity to non-cancerous tissue [1,2]. Liposomes are a versatile delivery system and modification of the liposomal surface with cell-binding ligands has been shown to facilitate active targeting, enhancing delivery and uptake in specific tissues [2–5]. Antibodies and peptides are frequently employed as targeting ligands and are commonly attached to liposomes through various chemical conjugation reactions [2,5–7], However, chemical modification of the liposomal surface with ligands can add complexity to the synthesis and increase the cost when scaling up the process [8].

An alternative approach to modify the surface of liposomes is through the use of spontaneously and remotely inserting membrane proteins. One example is the insertion of in vitro synthesised Bak, a tail-anchored (TA) protein that induces apoptosis, into preformed liposomes [9]. Bak-decorated liposomes have been shown to successfully induce apoptosis when incubated with cells in culture; upon delivery, individual Bak proteins spontaneously oligomerise within the cell membrane leading to permeabilisation and cell death. A more flexible approach is to construct targeted therapeutic proteoliposomes that have been decorated with an inert, purified, membrane protein containing an appropriate targeting sequence. The practicality of this method was demonstrated using a variant of the major coat protein PVIII from bacteriophage, which had been modified to display an incorporated tumour-targeting sequence within its N-terminal domain [10,11]. The chimeric protein was incorporated into drug-loaded liposomes, and the resultant proteoliposomes exhibited enhanced uptake by cells in culture. However, these liposomes suffered significant cargo leakage during the protein insertion reaction, because of its dependence on the presence of detergent [10,11]. Additionally, Kedmi et al. investigated the use of the NlpA lipophilic protein motif as an alternative anchoring system for the display of several antibodies on the liposome membrane [12]. In this study, we have explored an alternative mechanism for anchoring targeting peptides into liposome surfaces, by using a modifiable TA membrane protein chimera that is capable of spontaneous and remote insertion into the lipid bilayer of preformed, drug-loaded liposomes. Insertion occurs without inducing leakage of the liposomal payload, nor changing liposome size. Engineered TA membrane protein constructs provide the flexibility to incorporate different targeting motifs, providing the potential to create a library of selective tissue-targeting nanoparticles for use in personalised medicine.

**Fig 1:**
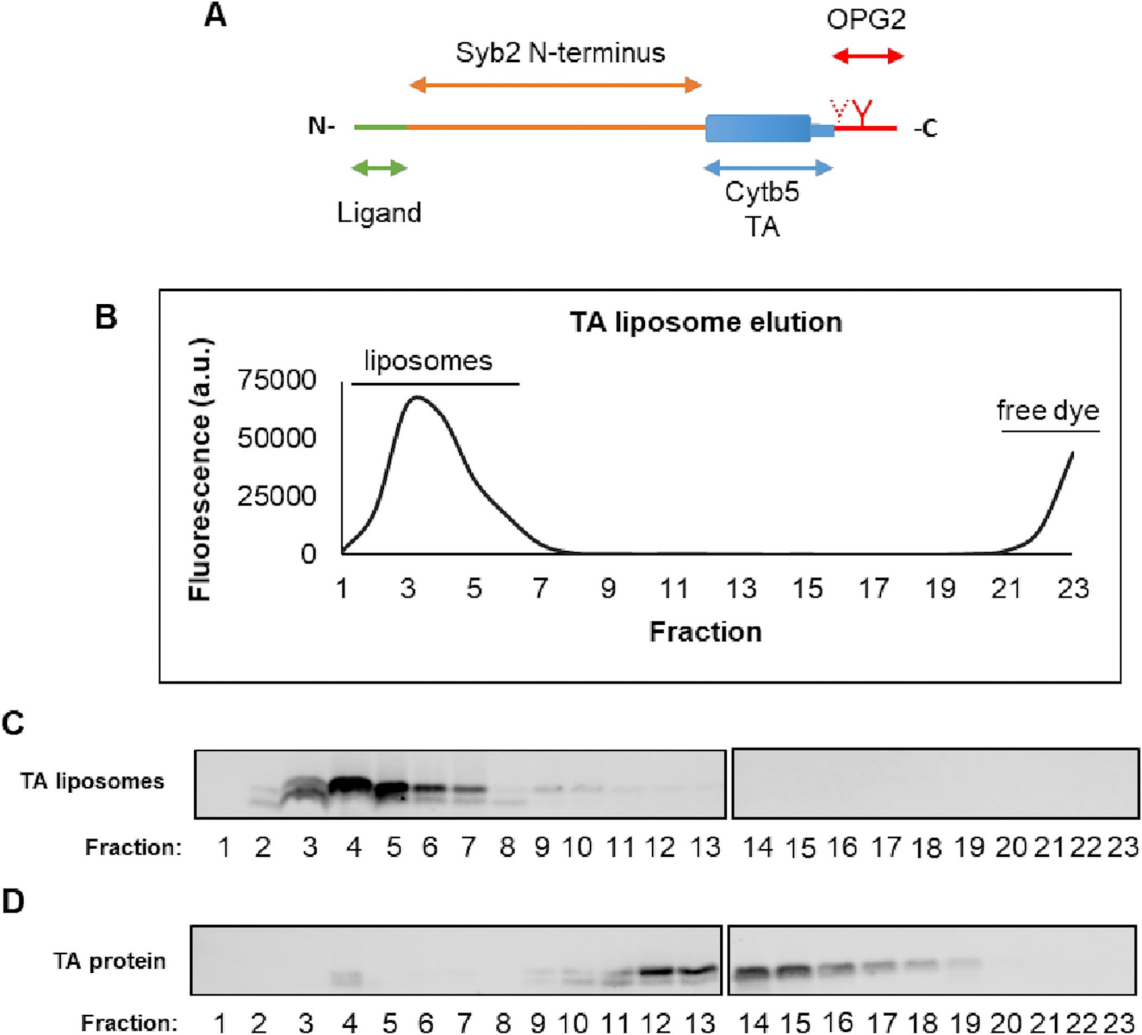
TA-proteoliposome production. The Syb2Cytb5OPG2 chimera was engineered to incorporate short peptide motifs immediately following the thrombin cleavage site at the N-terminus and a C-terminal opsin tag (OPG2) for the purpose of immunoblotting (**A**). Syb2Cytb5 (TA) was incubated with liposomes containing carboxyfluorescein (CF) and purified by size exclusion chromatography. The elution profile of the fluorescent cargo (**B**) was compared to that of the TA protein, detected by immunoblotting (**C**). The elution profile of free TA protein was analysed for comparison (**D**).

TA proteins constitute a subtype of membrane proteins characterised by a single membrane spanning region at or near the C-terminus [13]. This region functions both as a membrane anchor and a signal sequence recognised by chaperones responsible for delivering the TA proteins to their target membranes. Because of the C-terminal location of the tail anchor, post-translational mechanisms are employed for their membrane integration [13]. Different pathways are involved in the biogenesis of TA proteins and the exact pathway employed by any given TA protein appears to depend on the relative hydrophobicity of the TA region [13,14]. A handful of TA proteins such as cytochrome b5 (Cytb5) have been found to contain a TA region of relatively low hydrophobicity. This feature has been suggested to enable the spontaneous insertion of such TA proteins into both biological and synthetic bilayers, without the need for any additional components [15,16]. Interestingly, the TA region of Cytb5 has been shown to retain these properties when attached to non-native N-terminal domains [17,18]. In this study, we have investigated whether chimeric constructs containing the Cytb5 TA region can be employed to modify the surface of PEGylated liposomes with peptide-based ligands, in order to enhance their uptake by mammalian cells. The N-terminal domain of a second TA protein, synaptobrevin 2 (Syb2), was incorporated into the chimeric constructs to form a linker between the C-terminal Cytb5 TA region and selected targeting ligands (Fig 1A), to extend the availability of these ligands beyond the lipid-conjugated PEG moieties that are used to prolong the half-life of the liposomes [19,20]. Syb2 is part of the SNARE complex involved in intracellular vesicular fusion and should therefore not affect the binding of the liposomes to the cell surface [21]. An epitope tag (denoted OPG2), derived from the N-terminus of bovine opsin, was incorporated at the C-terminus of the chimeric construct to serve as a reporter for immunoblotting [17,22].

Three well established targeting ligands were employed in this study: GE11, TAT and CCGKRK. GE11 is a 12 residue peptide (YHWYGYTPQNVI) that binds to the epidermal growth factor receptor [23]. Decoration of liposomes with the GE11 sequence enhanced the efficacy of encapsulated doxorubicin in cell culture, and improved tumour accumulation and retention in animal models [24]. The trans-activator of transcription (TAT) peptide is a 9 residue (RKKRRQRRR) cell penetrating peptide that penetrates a wide range of cells via a mechanism that remains unclear [25–28]. The CCG ligand (CCGKRK) is derived from a targeting sequence suggested to bind the p32 receptor expressed on the cell surface of certain tumours; the same sequence has also shown selectivity towards placental tissue in mice [29–31]. In this study, we have utilised the Cytb5 TA to create customised targeted liposome formulations which demonstrate good stability, effective cargo delivery and enhanced cellular uptake.

## Materials and methods

### Materials

The pHisTrx expression vector was a gift from Richard Kammerer (formerly University of Manchester). The monoclonal antibody recognising the N-terminal region of bovine opsin used to detect the OPG2 tag is described in Adamus *et al*. (1991) [32]. 1,2-distearoyl-sn-glycero3-phosphocholine (DSPC), 1,2-distearoyl-sn-glycero-3-phosphoethanolamine-N[methoxy(polyethylene glycol)-2000] (PEG2000-PE) were purchased from Avanti Polar Lipids Inc (Alabama, USA). All other chemicals were acquired from Sigma-Aldrich (UK) unless otherwise stated.

### Recombinant protein expression and purification

Recombinant proteins were expressed in *Escherichia coli* strain BL21 (DE3) cells using Auto-Induction-Media (Formedium, Norfolk, UK). The cells were harvested and lysed in lysis buffer [50 mM Tris-HCl pH 7.5, 300 mM NaCl, 10 mM imidazole, 10% (v/v) glycerol, 5 mM 2-ME, 1 mM PMSF, 1 mg/ml lysozyme, 1 tablet of protein inhibitor cocktail/10 ml (Roche, West Sussex, UK) and 1% (w/v) n-dodecyl β-D maltoside (DDM)] for 1–2 hours in room temperature. Insoluble material was pelleted and soluble His-tagged TA protein chimeras then bound to nickel-NTA resin followed by extensive washing in buffer B [50 mM Tris-HCL pH 7.5, 300 mM NaCl, 10 mM imidazole, 10% (v/v) glycerol] supplemented with 0.1% (w/v) DDM, followed by a detergent exchange to 0.75% (w/v) n-octyl β-D glycopyranoside (OGP) as described by Leznicki *et al*. [33]. Additional washes in buffer B containing OPG were supplemented with the following additions: 0.05 mM ATP, 1 M NaCl and 0.5 M glycine. Finally, the beads were incubated with thrombin (15 U/ml) in buffer B containing 0.75% OPG overnight at room temperature [33]. Nickel-NTA resin was pelleted and 1 mM PMSF was added to the supernatant. Insoluble aggregates were pelleted by high speed centrifugation and the concentration of the purified TA protein estimated by its absorbance at 280 nm.

### Liposome and proteoliposome fabrication and characterisation

Liposomes were prepared using the thin film process. DSPC, DSPE-PEG2000 and cholesterol were dissolved in chloroform (molar ratio 60:5:35). Chloroform was removed by rotary evaporation and the resultant lipid film was hydrated using an appropriate buffer, as detailed below, resulting in a lipid concentration of 32.5 mM. Liposomes were extruded through polycarbonate membranes (Whatman, Schleicher & Schuell, Sigma-Aldrich) with the appropriate pore size using a mini extruder (Avanti Polar Lipids) at 60°C. For fluorescein containing liposomes, hydration was performed using 25 mM carboxyfluorescein (CF) dissolved in Dulbecco’s PBS (DPBS) followed by extrusion through 200 nm polycarbonate membranes. For doxorubicin containing liposomes, active remote loading of the drug was performed by first hydrating the lipid film in 250 mM ammonium sulphate (Merck, Southampton, UK), following a previously published protocol [34], followed by extrusions through 100- and 50 nm polycarbonate filters. In this study, fewer stacked filters were used so the resulting liposomes were larger than FDA-approved DOXIL, which has a mean diameter of 80nm. Extravesicular buffer was exchanged to 10% (w/v) sucrose using PD-10 desalting columns (GE Healthcare, Little Chalfont, UK), to create a pH gradient. The liposome preparation was incubated with doxorubicin (4mg/ml) (AdooQ BioScience, Irvine, CA, USA) at 60°C for 1 hour to achieve an encapsulation efficacy of >90%, as consistent with previous reports in the literature [34,35]. Extravesicular buffer was exchanged to DPBS by dialysis.

#### TA-protein modification

Liposomes (31 μM lipid) were incubated with TA protein in buffer B containing 0.75% (w/v) OGP) at 37°C for 90 minutes. One batch of liposomes was prepared with 3, 6 or 12 μM of TA protein; the liposomes used for all other experiments were prepared with 6 μM TA protein. The incubation time was based on data from previous stability studies which showed that there was no leakage of encapsulated cargo under these conditions [36]. The resultant proteoliposomes were purified by size exclusion chromatography (SEC) using CL-6B Sepharose resin. Encapsulated CF was detected by fluorescence at 490/520 nm (ex/em) after SEC; doxorubicin was detected by measuring the absorbance at 495 nm. Liposome size distributions were measured by dynamic light scattering using the Zetasizer Nano ZS (Malvern, Royston, UK).

#### Electron microscopy

3 μl of liposome solution (~10 mM lipid) was added to a carbon coated EM grid (carbon thickness ~5 nm) and removed after incubation at room temperature for 5 minutes. 50 μl 0.5% uranyl acetate was dropped onto the grid, in order to stain and wash the grid. Excess uranyl acetate solution was removed and the grid was dried prior to imaging. Images were acquired using a Tecnai BioTwin transmission electron microscope (FEI, Cambridge, UK).

### Flow cytometry

50,000 HeLa M cells/well or 75,000 BeWo cells/well were plated in a 12 well plate in complete medium. HeLa M were cultured in 6429 Dulbecco’s Modified Eagles Medium (DMEM) supplemented with penicillin (100 IU/ml), streptomycin (100 μg/ml), 2 mM glutamine and 10% (v/v) fetal bovine serum (FBS) (Gibco, Life Technologies, Warrington, UK). For BeWo cells, the complete media contained an equal mixture of 5796 DMEM and 6658 Ham’s F12 medium, supplemented with FBS and penicillin-streptomycin-glutamine as described above. The cells were incubated at 37°C for 23 hours, the medium was removed and replaced with serum-free medium, followed by incubation at 37°C for another hour. Medium was then replaced with 1 ml of serum-free medium containing CF-loaded liposomes (1 mM lipid). After 2 hours of incubation at 37°C, the liposomes were removed and cells were washed twice with DPBS, treated with trypsin (1x trypsin, Gibco, Life Technologies) and resuspended in FBS-containing medium. Cells were pelleted (4.2 g at 4°C) and resuspended in 400 μl DPBS containing 5 mM EDTA. Forward and side scatter was collected for samples using Accuri C6 flow cytometer (BD Biosciences, San Jose, CA, USA) and gated to record 10,000 events per sample reading in the FL1 filter (533/30 nm).

### Cell toxicity assays

#### MTT assay

2,500 HeLa M cells or 7,500 BeWo cells were plated in triplicate wells in a 96 well plate. Cells were placed in a humidified incubator with 200 μl complete media and incubated for 24 hours at 37°C. Media was replaced with 200 μl complete media containing purified liposomes, resulting in a reduction in the final concentration of doxorubicin to 50 μM. After a further 24 or 48 hours, media was removed and replaced with 50 μl complete media containing 0.5 mg/ml MTT. After an additional 3 hour incubation, 150 μl DMSO was added to wells and the plate was incubated at room temperature for 5 minutes. Metabolised MTT was resuspended by pipetting and the absorbance of the resulting solution was measured at 590 nm. The background signal measured at 670 nm was subtracted from the acquired absorbance readings at 590 nm. Cell viability was estimated by comparing the values obtained from the treated samples to those of the untreated control.

#### Caspase 3/7 assay

Cells were plated and treated as described above, but for this assay, the cells were seeded in 100 μl media in black 96 well plates with a clear bottom and were incubated for 48 hours prior to analysis. A caspase activity assay was conducted using the Amplite Fluorimetric Caspase 3/7 Assay Kit (AAT Bioquest, Sunnyvale, CA, USA) according to the manufacturer’s instructions.

### Data analysis

Data were analysed using Microsoft Excel 2013. Non-parametric data were expressed as means and analysed using standard deviations or one way analysis of variance (ANOVA) test. Data from a minimum of 3 independent experiments are presented. Significance was taken as P < 0.05.

## Results

### Creating targeted liposomes through TA modification

Liposomes loaded with carboxyfluorescein (CF) were fabricated, derivatised with different TA protein chimeras, and subjected to preliminary characterisation. The parental Syb2Cytb5 chimera was modified to display one of three different targeting ligands at its N-terminus (Table 1; Fig 1A). In the case of the CCG ligand, the sequence was duplicated in order to create a ligand of a similar length to the other two sequences employed (Table 1). All chimeric constructs contained two additional residues (GS) at their N-terminus, a result of the thrombin cleavage reaction employed during the protein purification process.

**Table 1:** Amino acid sequences of Syb2Cytb5 construct and ligands.

**Protein**	**Sequence after thrombin cleavage**	
Syb2Cytb5 OPG2	GS▪SATAATVPPAAPAGEGGPPAPPPNLTSNRRLQQTQAQVDEVVDIMRVNVDKVLERDQKLSELDDRADALQAGASQFETSAAKLKRKYWWKNLKWWTNWVIPAISAVAVALMYRLYMAEDSRMNGTEGPNFYVPFSNKTVC	39 80 121 141
**Ligand**	**Sequence**	
GE11	YHWYGYTPQNVI	12
CCG	CCGKRKCCGKRK	12
TAT	RKKRRQRRR	9

The sequence of the Syb2Cytb5 construct employed in this study is shown, along with the three sequences for the ligands. The Syb2Cytb5 sequence is shown after the removal of the N-terminal His-Thioredoxin tag by thrombin cleavage. The three ligands (see main text for full details) were inserted at the N-terminus of the Syb2 derived residues directly following the thrombin cleavage site (site of insertion indicated by ▪). The two residues ‘GS’ that remained at the N-terminus are underlined, as is the tail anchored region in the middle of the sequence, and the modified opsin tag (OPG2) located at the C-terminus. The sequence numbering is based on the native protein which excludes the ‘GS’ residues, while taking into account the endogenous N-terminal methionine residue.

**Fig 2:**
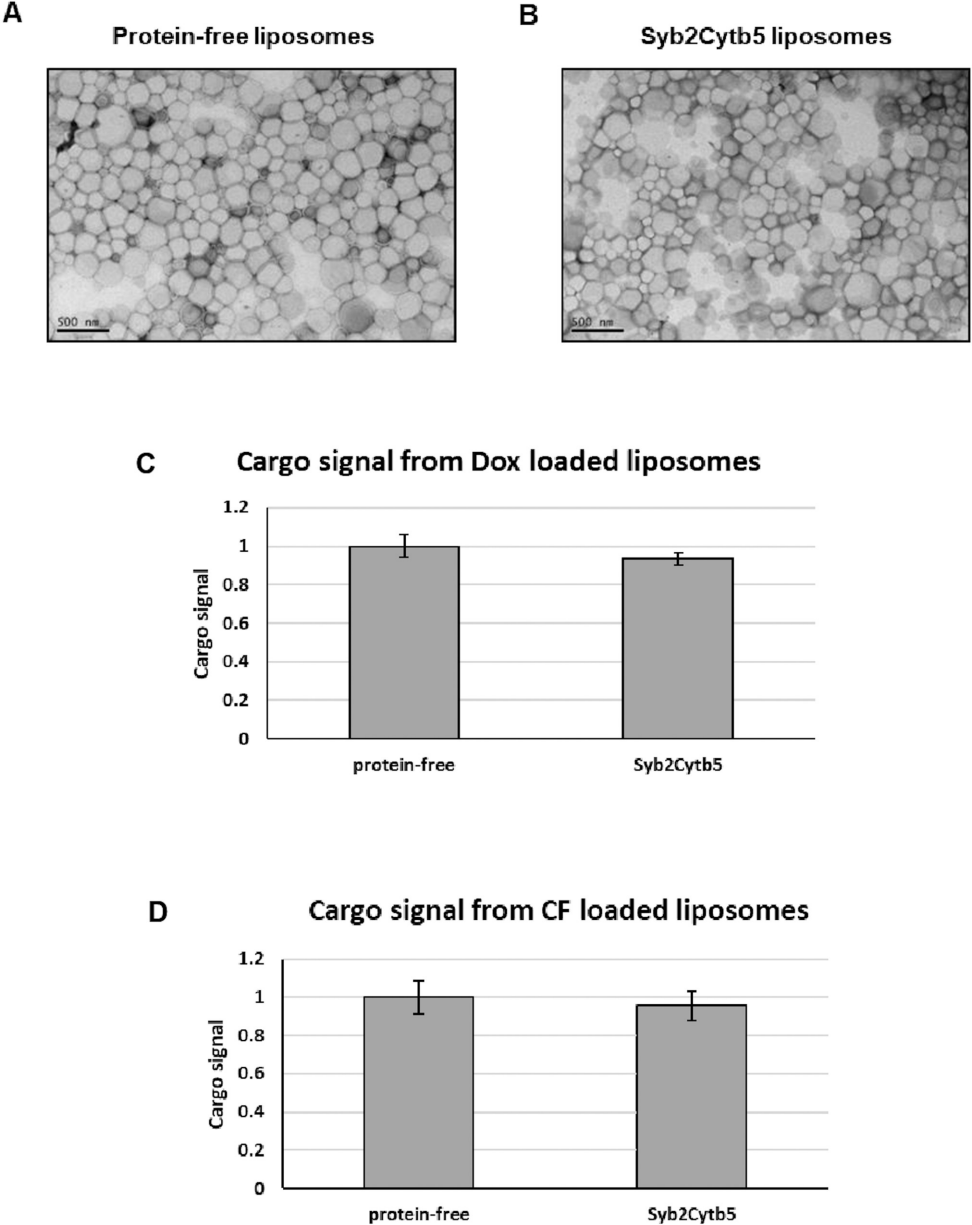
TA-proteoliposome characterisation. Liposome morphology was unaltered upon TA protein modification as imaged by transmission electron microscopy (**A** and **B**). Scale bar = 500nm. CF cargo in liposomes appears to be unaffected by TA incorporation (**C**): Total absorbance of the Dox-cargo was measured for the different preparations as indicated. The values represent the mean absorbance ± standard deviation (n = 3), and are normalised to the signal from protein-free liposomes (n = 1). (**D**): Total fluorescence from the CF cargo measured for the separate preparations. The values are shown as the mean fluorescence ± standard deviation (n = 3) and normalised to the mean protein-free liposome signal (n = 1). The Dox-cargo appears to be substantially retained upon TA modification.

**Fig 3:**
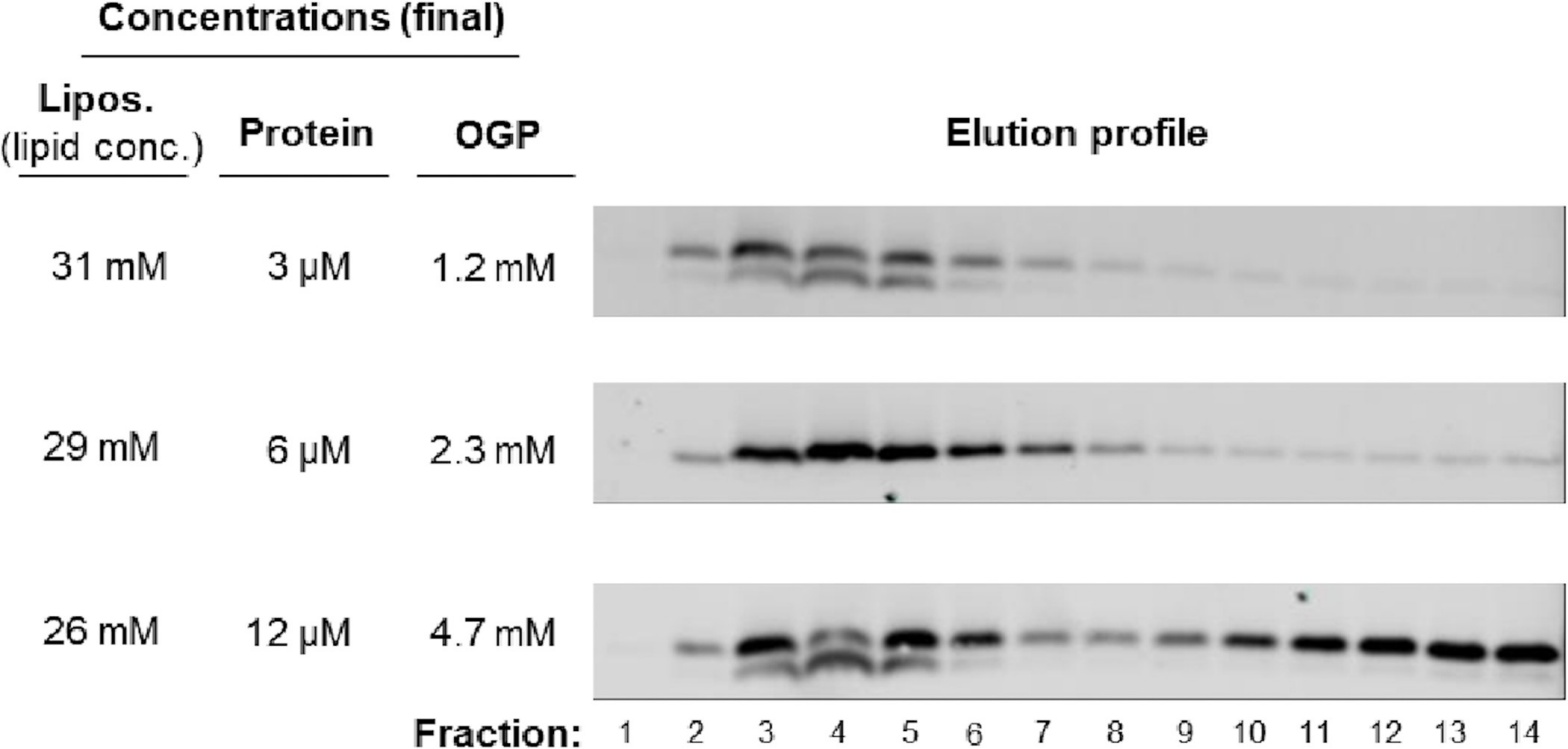
TA-protein insertion into Dox-containing liposomes is saturable. TA-protein (Syb2Cytb5) in buffer containing octyl β-D-glucopyranoside (OGP) was incubated with liposomes at the specified concentrations. After incubation, the reactions were loaded onto a SEC column for purification. The fractions were collected and protein content was detected by immunoblotting for the C-terminal OPG2 epitope.

Liposomes used in drug delivery usually consist of saturated lipids, PEGylated lipids and a relatively high percentage of cholesterol. These liposomes are significantly more rigid than those derived from unsaturated lipids, which have previously been used to study the spontaneous insertion of the Cytb5 TA into liposomal membranes [15,16,37]. Previous attempts to rigidify these unsaturated lipid bilayers by the inclusion of cholesterol significantly inhibited the incorporation of the Cytb5 TA [16]. In order to overcome this problem, we elevated the temperature that we employed during the TA protein insertion reaction to 37°C. The co-elution of the Syb2Cytb5OPG2 proteins with the liposome-encapsulated CF on a size exclusion column suggests that the TA protein was successfully membrane incorporated (Fig 1B and 1C). In comparison, the free TA protein was eluted in later fractions (Fig 1D). Electron microscopy images of the CF-loaded liposomes confirmed that their gross morphology remained unchanged after the insertion of TA proteins into the liposomal membrane (Fig 2A and 2B). The mean diameter of all CF-loaded liposome preparations lay between 178–185 nm, and the mean polydispersity index (PDI) for each preparation was <0.06, indicative of a highly homogeneous unimodal size distribution (Table 2). The mean fluorescent signals detected from the encapsulated CF and DOX cargoes were comparable between preparations and we found no evidence of any significant leakage from the liposomes as a consequence of TA protein insertion into the liposome bilayer (Fig 2C and 2D). Ligand density was estimated to be approximately 25 copies of Syb2Cytb5OPG2 per liposome, using the calculation described by Guven *et al*. [38]. Experiments titrating the TA-protein concentration confirmed that ligand insertion into Dox-containing liposomes was saturable (Fig 3).

**Table 2:** Liposome size is not affected by association with TA protein.

Liposome preparation	CF-loaded		Dox-loaded	
	Diameter (nm ± SD)	PDI	Diameter (nm ± SD)	PDI
Protein-free	181.9 ± 3.1	0.051	133.2 ± 2.6	0.035
Dox-free	-	-	133.9 ± 0.7	0.045
Syb2Cytb5	180.1 ± 3.7	0.044	134 ± 2.8	0.037
GE11 Syb2Cytb5	178.7 ± 3	0.057	135.1 ± 2.9	0.035
TAT Syb2Cytb5	179.1 ± 2.9	0.033	133.7 ± 1.9	0.033
CCG Syb2Cytb5	184.9 ± 2.6	0.05	134.6 ± 2.4	0.034

Mean diameter of different liposome formulations, was determined using dynamic light scattering. Mean polydispersity index (PDI), a measure of homogeneity of the size distribution is also reported. CF, carboxyfluorescein; Dox, doxorubicin; PDI, polydispersity index; SD, standard deviation. (n = 3).

### Uptake of targeted TA modified liposomes in human cell culture

**Fig 4:**
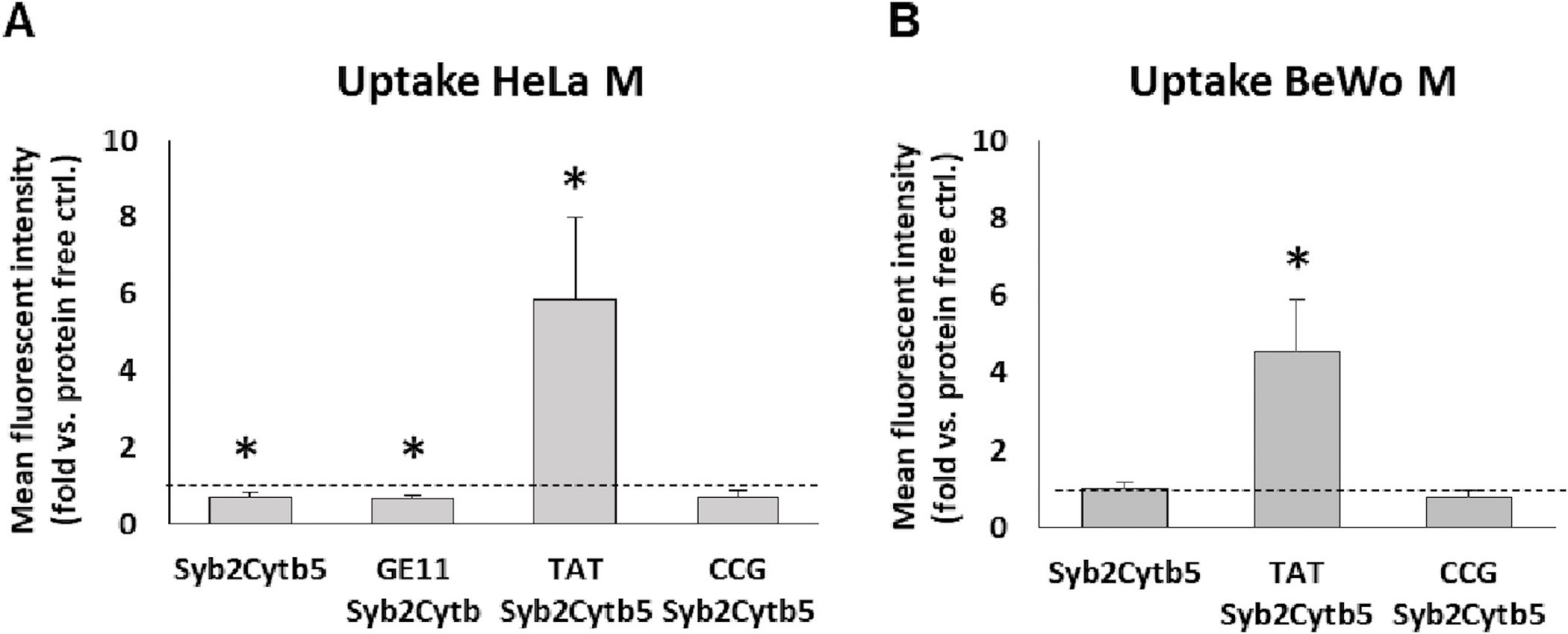
Effect of distinct peptide ligands on cargo uptake by cultured cells. Liposomes modified with TA proteins were incubated with HeLa M (**A**) and BeWo (**B**) cell lines in serum free medium for 2 hours. The cell associated fluorescence derived from internalisation of liposome cargo was measured by flow cytometry. A complete set of ligands were investigated with HeLa M cells (n = 5), while BeWo cells were exposed to a subset of ligand-decorated liposomes (n = 3). Mean fluorescence intensity of samples relative to protein-free liposomes (dashed line) is reported along with the standard deviations. Significance of each signal relative to the protein-free liposome treatment was measured by one-way ANOVA test, p < 0.05 is indicated (*).

CF-loaded liposomes modified with the TA chimeras outlined in Table 1 were incubated with two different human cell lines for 2 hours and the extent of CF delivery was quantified by flow cytometry. The placentally-derived choriocarcinoma cell line BeWo was selected, to take advantage of the previously reported placental homing property of the CCG ligand [31]. HeLa M cells were chosen as they reportedly express the cognate receptors for both the GE11 and CCG ligands [39,40]. The same cognate receptors are also expressed by human placental cytotrophoblasts [41,42]. Fluorescence intensity following proteoliposome uptake was normalised to the signal detected in cells incubated with protein-free liposomes. The level of fluorescent signal detected in HeLa M cells following incubation with liposomes modified with the parental Syb2Cytb5 showed a modest decrease, when compared to that of cells incubated with protein-free liposomes (Fig 4A). Likewise, a decrease in signal of ~30% was observed with liposomes bearing the Syb2Cytb5 chimera modified with the GE11 ligand (Fig 4A). In comparison to these two chimeras, the inclusion of the TAT ligand resulted in a striking 5.8-fold increase in the level of cell-associated fluorescence, whilst the CCG ligand did not alter the signal as compared to control protein-free liposomes (Fig 4A). When a subset of these liposomes were analysed using BeWo cells, only the proteoliposome preparation that incorporated the TAT ligand resulted in an increased CF signal after 2 hours, which was again significantly higher than the control (Fig 4B).

### Effect of TA-mediated ligand modification of liposomes on doxorubicin delivery

**Fig 5:**
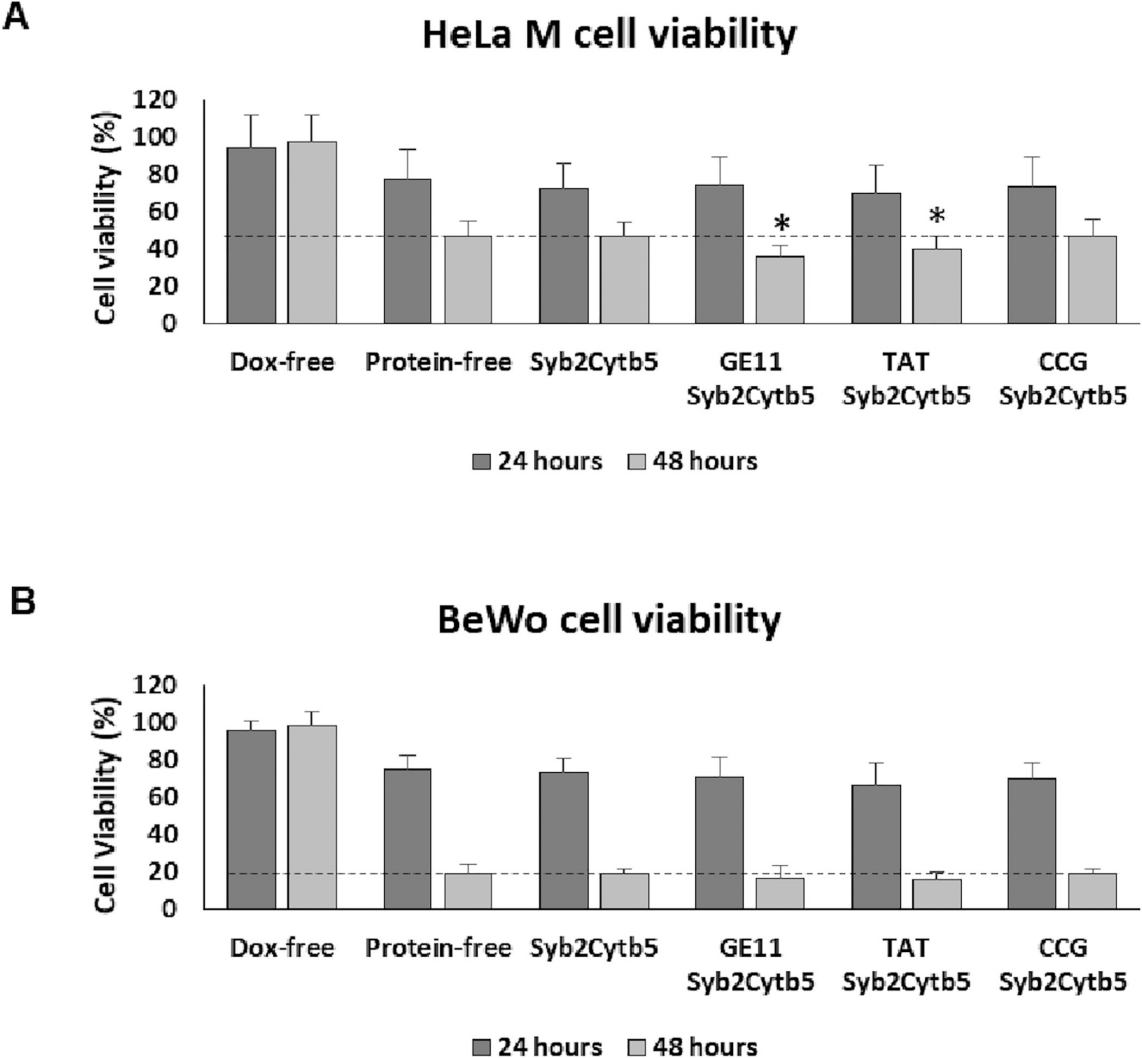
Effect of different TA chimeras on liposome mediated cell toxicity. HeLa M (**A**) and BeWo (**B**) cells were incubated with liposomes in triplicate for 24 and 48 hours followed by incubation with MTT for 3 hours. Cell viability is shown as the percentage of the total MTT signal detected in untreated control cells. Error bars corresponds to the standard deviation (n = 4). Significant changes in cell viability (* indicates p < 0.05) compared to protein-free liposomes (see dashed line for 48 hour time point) was determined using One-way ANOVA test.

Liposomes remotely loaded with a doxorubicin (Dox) cargo were employed in order to study the effect of ligand attachment on the delivery of an established chemotherapeutic agent. Dox-loaded liposome batches were uniform in size (mean diameter 133–135 nm; mean PDI <0.045); regardless of TA-protein modification (Table 2). Empty liposomes (Dox-free) and unmodified Dox-loaded liposomes (protein-free) were employed as controls. For these experiments, HeLa M and BeWo cells were incubated with the full range of liposome preparations for 24 or 48 hours. Cell viability, as measured by MTT assay, was not significantly different from the Dox-loaded, protein-free control values in HeLa M (Fig 5A) or BeWo cells (Fig 5B) after treatment for 24 hours with any of the proteoliposome formulations. After 48 hours, the percent of viable HeLa M cells was reduced by 8.5% following treatment with proteoliposome preparations displaying either the GE11 or TAT ligands (Fig 5A), as compared to the Dox-loaded, protein-free control liposomes. At this later time point, BeWo cells were more sensitive to all of the liposomal formulations containing Dox, when compared to the HeLa M cells. However, none of the individual ligands evoked a significant decrease in BeWo cell viability compared to that observed following treatment with Dox-loaded, protein-free liposomes (Fig 5B).

**Fig 6:**
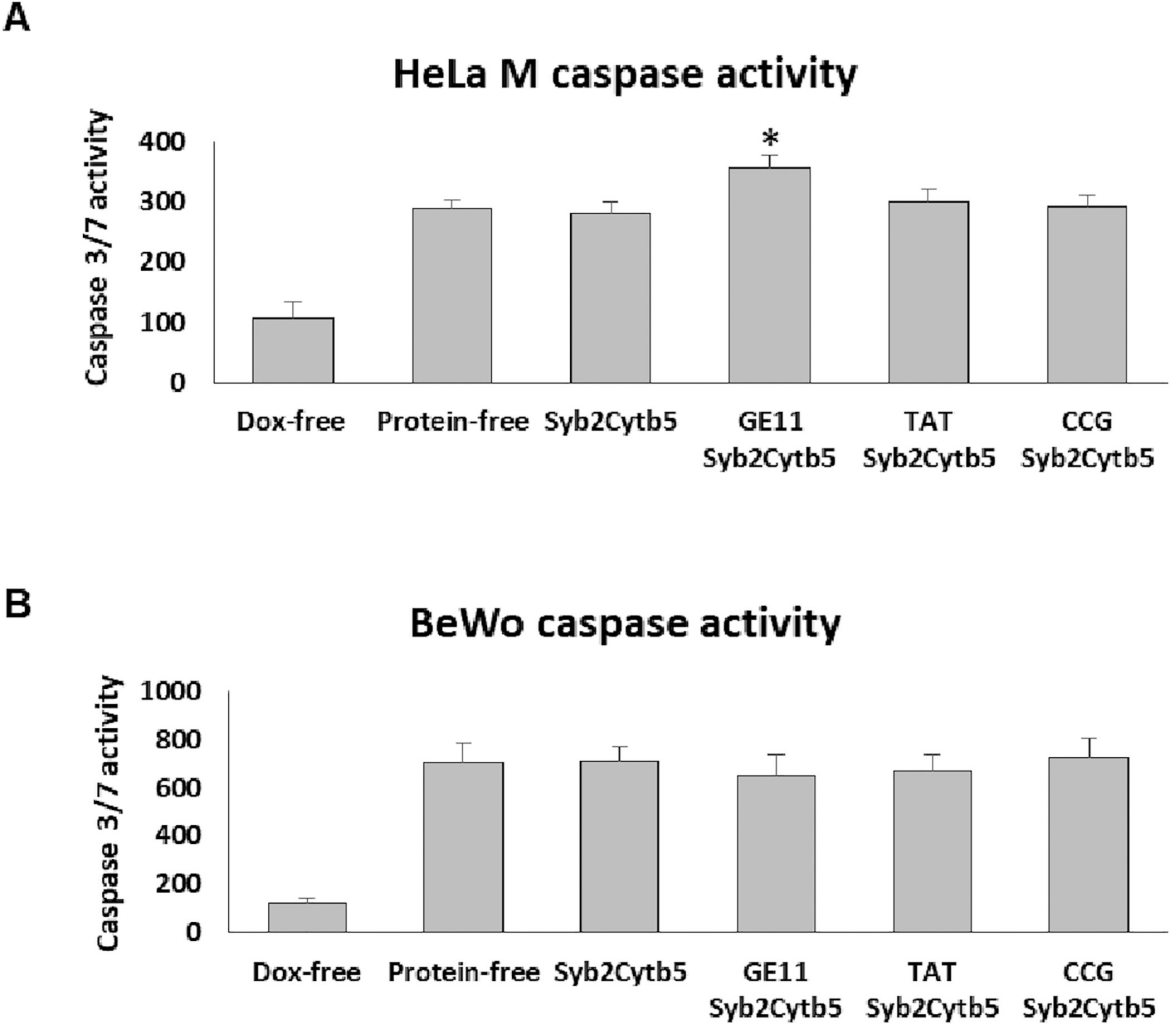
Effect of TA chimeras on liposomal Dox-mediated activation of caspase 3/7. HeLa M (A) and BeWo (B) cells were incubated with Dox-loaded liposomes with or without incorporated TA proteins as indicated. The activity of caspase 3 and caspase 7 was measured after 48 hours of incubation. The values shown are expressed as a percentage of the value obtained using untreated cells. Error bars correspond to the standard deviation (n = 3) and the significance of the acquired values relative to protein-free Dox-loaded liposomes was determined by one-way ANOVA test (* indicates p < 0.05).

The cytotoxic effect of the Dox-loaded liposome preparations was investigated further by monitoring the extent of apoptosis in each cell type using a caspase 3/7 activity (Fig 6) [43]. Caspase 3/7 activity increased following treatment of both cell lines with all Dox-loaded liposome preparations for 48h, when compared to treatment with Dox-free liposomes (Fig 6). However, in HeLa M cells, the GE11 ligand induced an additional significant increase in caspase activity, compared to that observed following treatment with Dox-loaded, protein-free liposomes (Fig 6A). In BeWo cells, none of the ligands increased the level of caspase activity above that observed following treatment with the Dox-loaded protein-free liposomes, consistent with the results obtained using the MTT assay (Figs 5B and 6B).

## Discussion

In the present study, an alternative method for the display of ligands at the surface of liposomes without the need for chemical coupling was developed. This method exploits the TA of Cytb5 as an anchoring platform for N-terminally fused ligands. The ability for spontaneous membrane incorporation appears to be solely retained within the Cytb5 TA, since it has been shown to be capable of incorporating non-native N-terminal domains into lipid bilayers [17,18]. Here, we use the Syb2 N-terminal domain as a linker between the Cytb5 TA and three different ligands that have been demonstrated to significantly enhance the uptake of liposomes by their cellular targets [24,27,31].

A previous study suggested that cholesterol inhibits the spontaneous insertion of TA proteins into synthetic membranes when present at concentrations as low as 5 mol% [16]. Our study shows that this potential incompatibility with cholesterol-containing membranes can be overcome by elevating the reaction temperature to 37°C during membrane insertion. This most likely increases the fluidity of the liposomal membrane, allowing the TA protein to more easily insert, making it possible to incorporate TA protein chimeras into liposomes containing 35 mol% cholesterol, a comparable level to that of DOXIL. Moreover, this study demonstrates the insertion of TA proteins into a PEGylated liposomal system for the first time, a significant finding given the importance of cholesterol and PEG in the preparation of therapeutic liposome preparations [44,45].

To prepare TA-modified liposomes, our chimeric membrane proteins could be incubated with preformed liposomes without any need for detergent beyond the small amount of OGP (final conc: 1.2–2.3 mM) that was used during the purification of the TA-protein chimeras [35]. We estimate that the liposomes become saturated with TA-proteins (cf. Fig 3) at a relatively low density (~ 25 copies / liposome)[38], as compared to the several thousand ligands per liposome that may be achieved via direct coupling methods [24,31]. However, the comparable amount of Dox present in TA-modified liposomes suggests that the incorporation of TA-protein chimeras does not result in any significant drug leakage (Fig 2D). In contrast, two studies that employed detergent to facilitate the integration of membrane proteins into liposomes observed cargo leakage in the range of 30–95% for encapsulated Dox [10,11].

In the present study, the effect of TA incorporation on liposome size was measured by dynamic light scattering and the potential effects on liposome morphology were studied through negative stain electron microscopy. The results showed that there was no significant alteration to the size of the liposomes after TA incorporation and that the liposomes remained similar in appearance upon TA modification; however, Dox loaded liposomes were smaller (~133nm diameter) than those loaded with CF (~180nm diameter) The encapsulation efficiency of CF was not altered upon modification with the TA proteins.

When TA modified liposomes were incubated with two different cell lines, the inclusion of the N-terminal domain of Syb2 alone did not significantly influence liposome uptake (Table 1), confirming it provides an appropriate and neutral linker for targeting peptides. Of the three ligands added to the Syb2 linker region in this study, only the cell penetrating TAT sequence significantly enhanced delivery of the CF cargo to both cell lines after 2 hours, indicative of a short term increase in the rate of liposomal uptake. Although the exact mechanisms remain unknown, the TAT sequence is rapidly internalised into numerous cells types [46,47], at a rate reported to be much faster than known endocytic processes [48]. As such, it likely accumulates at a faster rate than the other two ligands; GE11 and CGKRK mediate internalisation by the classical endocytosis pathway [49,50]. Alternatively, the failure of the GE11 and CCG sequences to enhance CF uptake may reflect that only low levels of the TA protein chimeras were incorporated into the liposomes via spontaneous insertion. Whereas our present method of attachment results in ~25 ligands per liposome, chemical coupling methods can attach ~2,000 to 10,000 copies of the same ligands per liposome. The substantial increase in CF signal observed using the TAT peptide is consistent with evidence that as little as five copies of this ligand per liposome can enhance uptake [28]. This proof of concept study has demonstrated enhanced uptake of liposomes <200nm in diameter. As liposomal surface area is increased proportionally as diameter is reduced, efficacious TA protein insertion should theoretically be observed with smaller therapeutic liposomes such as DOXIL, which has a mean diameter of 80nm.

The enhancement in cell-associated CF observed after 2 hours of incubation with TAT-decorated proteoliposomes did not translate into a substantial increase in cell death at 24h or 48h, when a cytotoxic Dox cargo was employed. Whilst both the TAT and GE11 ligands effected a small but significant reduction in the viability of HeLa M cells, only the GE11 ligand modestly enhanced the level of caspase-3/7 activity at 48h, indicative of apoptosis. These differences may be due to the experimental model used; in the closed system of a cell culture, any therapeutic benefit of a short term enhancement of drug delivery may be lost during extended culture periods, when enough time has passed for protein-free liposomes to deliver an equivalent payload to the target cells. Exploring the time course of cell death in more detail would elucidate the kinetics of uptake, which is most likely faster than initially hypothesised. However, another study has proposed that the failure of the TAT peptide to deliver a cytotoxic payload was due to its uptake via a mechanism that does not allow the associated liposomal cargo access to the nucleus [28]. If this is indeed the case, then this issue may be circumvented by the incorporation of additional fusogenic agents, such as the influenza hemagglutinin peptide [51], into the liposome membrane and/or by using alternative high efficiency ligands that employ different pathways for cell entry [52]. For instance, the antibody ligand RI7217, used to target cells within the brain, was shown to be efficient in delivering a liposome cargo in both cell culture and an animal model, when present at a level of 20–25 copies of antibody per liposome [52].

Other features that may influence the effectiveness of TA chimeras for enhancing liposomal drug delivery in future studies include the size and nature of the linker employed. A previous study using membrane anchors to modify liposomes with targeting ligands successfully employed a 16 amino acid long linker region [10], significantly shorter than 95 residue linker that we employed here. Likewise, alterations to the liposomal formulation may enhance ligand attachment, particularly given rigidifying components can reduce the efficiency of spontaneous insertion for TA proteins [15,16]. Similarly, liposome diameter may affect the mechanism by which they are internalised [53,54], and it appears that smaller liposomes (< 100 nm) are more readily internalised by clathrin, dynamin or caveolar-dependent endocytosis [53]. Smaller liposomes may therefore benefit from the use of targeting ligands, such as GE11, that induce uptake through clathrin-mediated endocytosis [24]. It is also important to note that the targeted liposome preparations used here may demonstrate a more pronounced degree of tissue selectivity if administered to tissue explants or animal models [24,31].

In summary, the results from the present study show that the use of the Cytb5 TA to anchor ligands into liposome membranes is a viable alternative to chemical coupling. However, the potentially smaller number of TA protein chimeras that can be incorporated into the liposomes means that this method may only be compatible with high efficiency ligands that can increase cellular uptake even when displayed in low numbers. The lack of enhanced cell delivery in vitro compared to protein-free liposomes indicates that further advancements are required for this approach to be viable. This will include increasing the number of ligands displayed per liposome and confirming an enhancement of uptake for encapsulated cytotoxic drugs. In an optimised form, this system could provide a convenient and scalable “one-step” process for the tailored modification of “off the shelf” liposome preparations that use one of a range of targeting ligands designed to enhance their selective delivery.

## Conclusion

The spontaneously inserting TA region of Cytb5 is capable of anchoring N-terminal domains incorporating cell binding ligands into liposomes. Modification with specific TA protein chimeras variably enhanced the uptake of liposomes containing fluorescent and/or cytotoxic cargoes in human cell culture models. We conclude that the Cytb5 TA presents an exciting opportunity to anchor a range of polypeptides onto the surface of synthetic lipid bilayers, and that with further optimisation this approach may be suitable for enhanced drug delivery.

## Supporting information

S1 Fig Uncropped western blots. Uncropped versions of the wester blots shown in Fig 1C (**A1, 2**), Fig 1D (**B1, 2**) and Fig 3C–3E.
(TIFF)
