# Supplementary figures and images for: The use of tail-anchored protein chimeras to enhance liposomal cargo delivery
Source: PLoS One. 2019 Feb 22;14(2):e0212701. doi: 10.1371/journal.pone.0212701 (PMC6386398; doi:10.1371/journal.pone.0212701)

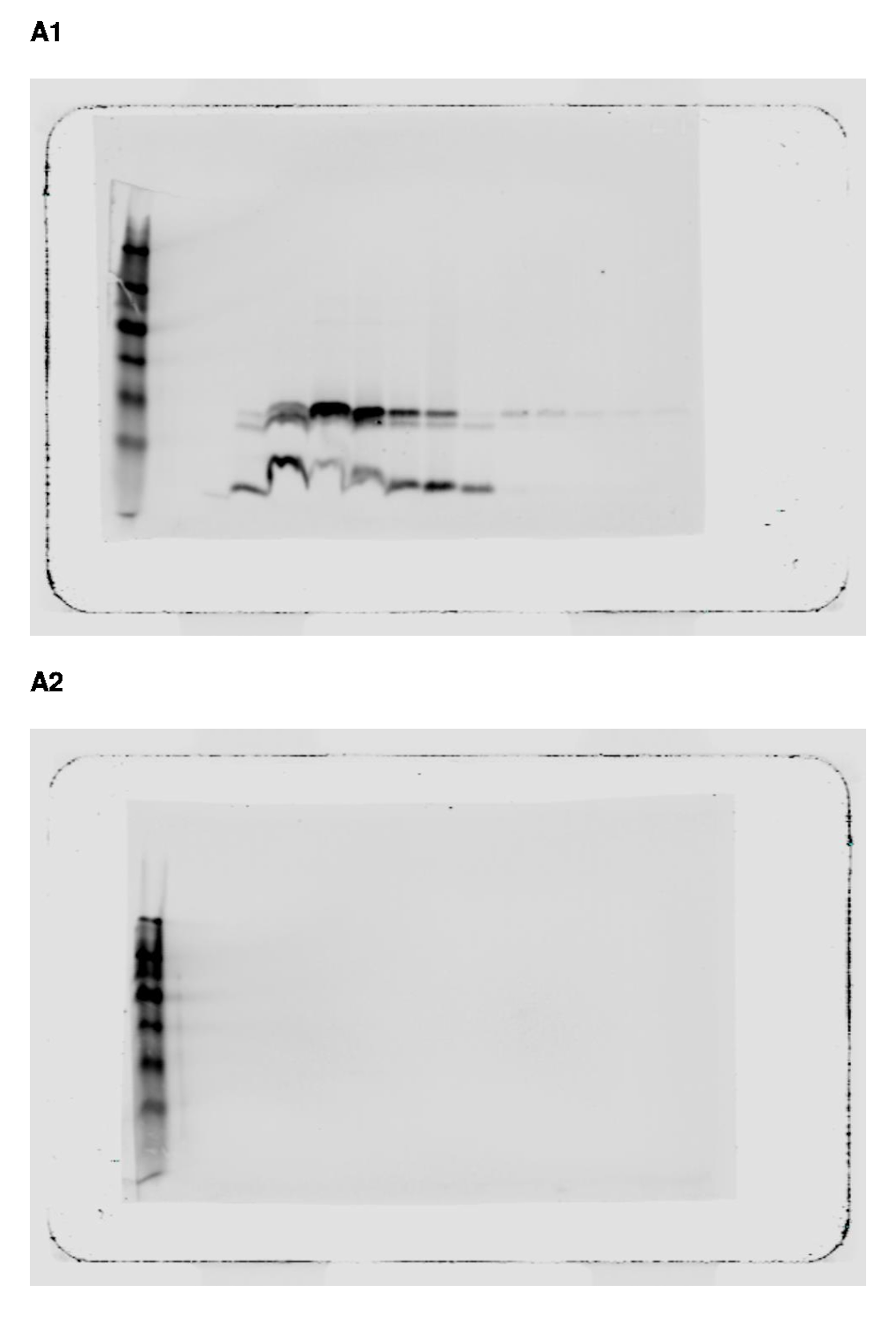

Supplement: S1 Fig — Uncropped versions of the wester blots shown in Fig 1C (A1, 2), Fig 1D (B1, 2) and Fig 3C–3E. (TIFF) [file pone.0212701.s001.tiff]
